# Do Salivary *Cullin7* Gene Expression and Protein Levels Provide Advantages over Plasma Levels in Diagnosing Breast Cancer?

**DOI:** 10.3390/cimb47010019

**Published:** 2024-12-31

**Authors:** Ceren Tilgen Yasasever, Derya Duranyıldız, Süleyman Bademler, Hilal Oğuz Soydinç

**Affiliations:** 1 Department of Basic Oncology, Institute of Oncology, Istanbul University, Istanbul 34093, Turkey; deryady@istanbul.edu.tr (D.D.); hoguz@istanbul.edu.tr (H.O.S.); 2 Department of General Surgery, Faculty of Medicine, Istanbul University, Istanbul 34093, Turkey; suleyman.bademler@istanbul.edu.tr

**Keywords:** breast cancer, Cullin7 (Cul7), saliva, plasma, protein, gene expression

## Abstract

In addition to the tumor suppressor role of Cullin 7 (Cul7), one of the proteins belonging to the Cullin (Cul) family, studies have also suggested that Cul7 may act as an oncogene under certain conditions. The role of the Cul7 molecule in breast cancer is still unclear, and understanding its function could have significant implications for identifying novel therapeutic targets or improving diagnostic strategies in breast cancer management. In this study, the levels of the Cul7 molecule in plasma and noninvasive material saliva were investigated, and its possibility as a marker for breast cancer was discussed. Protein levels of blood and saliva samples taken from breast cancer patients and a healthy control group were measured by the ELISA (Enzyme-Linked Immunosorbent Assay) method. Gene expression levels between the two groups were analyzed by the qPCR (quantitative Polymerase Chain Reaction) method. In our study, *Cul7* mRNA and protein expression levels were examined in 60 breast cancer patients and 20 healthy female controls, and a statistically insignificant difference was found between the patient and control groups in both plasma and saliva samples (*p* > 0.05). No correlation was found between the clinical characteristics of the patients and plasma and saliva *Cul7* gene expression and protein levels (*p* > 0.05). Considering the possibility of Cul7 being a biomarker at the protein and mRNA levels, plasma is thought to be a better study material for Cul7. Our findings suggest that in the context of a study on salivary material, the expression of *Cul7* at the mRNA level may have better potential utility as a biomarker.

## 1. Introduction

Breast cancer remains the most frequently diagnosed cancer today, with an estimated 2.3 million new cases per year, and is among the leading causes of cancer death among women worldwide [1]. The highest incidence rates are seen in Australia, New Zealand, Western Europe, North America, and Northern Europe, while the lowest incidence rates are seen in Central America, East and Central Africa, and South-Central Asia [2]. These rates are associated with a higher prevalence of reproductive and hormonal risk factors (later menopausal age and having fewer children, etc.) [3], lifestyle risk factors (alcohol intake [4], excessive body weight [5], physical inactivity [6], etc.), and increased diagnosis due to mammographic screening. Previous research has identified molecular biomarkers such as CA15-3 (cancer antigen 15-3), uPA (Urokinase-type plasminogen activator), and HER2 (Human Epidermal Growth Factor Receptor 2) as being associated with breast cancer. These biomarkers have been developed and utilized for early-stage detection, adjunctive diagnosis, and evaluation of prognosis [2,7]. Although biomarkers such as CD24 (Cluster of differentiation 24), TACC3 (Transforming acidic coiled-coil), and CIP2A (Cellular Inhibitor of PP2A) have been recently defined to predict the prognosis of breast cancer, timely diagnosis and effective treatment remain challenging due to the high heterogeneity and rapid progression of the disease. Today, this disease is the most common cancer in women and alone accounts for 25% of cancer cases and 15% of cancer-related deaths in women. Therefore, it is urgent to develop new treatments for breast cancer, to more accurately predict prognosis, to diagnose cancer, and to identify a biomarker to elucidate the pathways responsible for breast cancer metastasis [8,9,10,11].

Proteins belonging to the Cullin (Cul) family have critical roles in cancer, but few studies have been reported on Cul7 due to its characteristic molecular structure [12]. Cul7 forms a complex with ROC1 (Ring-box 1, Rbx1) ring finger protein, while only two Fbox proteins, Fbxw8 and Fbxw11, have been shown to bind to Cul7 [12]. Distinctly, Cul7 selectively interacts with the Skp1-Fbxw8 heterodimer rather than the monomeric Skp1 protein that is typically seen in SCF-type E3 ligases [13]. Interestingly, Cul7 can interact with its substrates by forming a new complex independent of these two F-box proteins. Cul Ring Ligase 7 (CRL7) is a novel complex composed of Cul7, Skp1-Fbxw8, and Rbx1. Due to the biological effects of CRL7, it is thought that it may not only have a proteolytic function but also play a non-proteolytic role [12]. While studies show that Cul7 has tumor-suppressive effects, there are also studies suggesting the opposite [12]. *Cul7* is highly expressed in many malignant tumors, including lung, liver, breast, and ovarian cancers, and its expression is known to be closely related to clinical staging and prognosis [12].

The mechanism of Cul7 in cancer still remains unclear because Cul7 plays a role in cell proliferation and invasion by regulating cell cycle and microtubule stability. Studies have reported that silencing *Cul7* expression reduces cyclin A but increases p21 protein expression. It has even been reported that silencing *Cul7* expression changes cell shape, causes microtubule disorder, and increases microtubule regeneration in MDA-MB-231 and BT549 cells. It is thought that it can be used as a new biomarker for early detection and treatment of breast cancer, and targeting CRL7 may be an effective strategy for cancer prevention and treatment [12,14]. Based on this information, we aimed to determine the gene expression and protein levels of the Cul7 molecule in plasma and saliva samples of patients with breast cancer.

## 2. Materials and Methods

### 2.1. Patient Sample Collection and Processing

This study included 60 female patients who were diagnosed with breast cancer clinically, radiologically, and pathologically, over 18 years old, who applied to Istanbul University Oncology Institute between 2018 and 2022, and 20 healthy volunteer women over 18 years old, not previously diagnosed with cancer, as the control group. All samples of the patients were collected before any treatment was administered. The mean age of breast cancer patients was 49.06 (27–77), and the mean age of healthy female control group was 40.45 (36–56). Age, ER (Estrogen Receptor), PR (Progesterone Receptor), HER2, histological type, and menopause information of 60 breast cancer patients are given in Table 1.

Blood samples of the patient and control groups were obtained by venipuncture and clotted at room temperature. Plasma samples (BD Vacutanier K2 EDTA 7.2 mg, Becton, Dickinson and Company, New Jersey, United States of America) were collected following centrifugation (10 min, 4000 rpm; Hettich Universal 32) and immediately frozen at −80 °C until analysis. Saliva samples taken from patients in sterile tubes (no preservative or stabilizer added) were centrifuged and stored at −80 °C until testing. The samples were taken in the morning after rinsing the mouth with water, and the patients were warned not to consume food or drink for at least an hour beforehand [15].

Our study was approved by the Istanbul University Ethics Committee (7 February 2020, no: 291). The ethical approval explicitly included provisions to ensure data protection and confidentiality. These provisions anonymized and securely stored all patient data. The protocol was consistent with the Declaration of Helsinki (1989). Informed consent was obtained from all the study participants.

### 2.2. Determination of the Gene Expression Levels

#### 2.2.1. RNA Isolation

An amount of 0.4 mL of the samples was placed in the tubes, and 1 mL of RNAzol©RT (Molecular Research Center, Inc., Cincinnati, OH, USA) was added. Then, 0.4 mL of water was added to the obtained homogenate, rested for 5–15 min, and centrifuged at 12,000× *g* for 15 min (Tehtnica Centric 200R, DOMEL, d.o.o., Železniki, Slovenia). The supernatant obtained at the end of centrifugation was transferred to another tube, and 1 mL of 75% ethanol was added and left for 15 min. Afterward, centrifugation was carried out for 10 min at 12,000× *g*. After this process, the liquid in the tube was discarded, and 0.4 mL of 75% ethanol was added to the white pellet at the bottom and centrifuged at 4000× *g* for 1–3 min. This process was repeated twice. After centrifugation, the alcohol was removed with a pipette without damaging the pellet. It was dissolved in water without drying the RNA pellet and vortexed for 2–5 min to make the dissolution process more effective. The measurements of isolated RNAs were performed with Nanodrop (Nanodrop: 1000; Thermo Fisher Scientific, Wilmington, DE, USA).

#### 2.2.2. Complementary DNA (cDNA) Synthesis

After the total RNA was obtained, the Jena Bioscience Script cDNA synthesis kit was used for cDNA synthesis (Jena Bioscience GmbH, Jena, Germany). All stages were carried out on ice. All RNA concentrations were equalized before the cDNA synthesis. The reaction was prepared with a total volume of 20 µL for each example (Table 2). This mixture was distributed to the wells, and then, the RNA products were added. Then, the sample mixtures put into the plate were placed on the Biorad CFX Connect apparatus. cDNA generation was completed by incubation at 42 °C for 10 min, 50 °C for 60 min, and 70 °C for 10 min. The resultant cDNA samples were stored at −20 °C.

#### 2.2.3. Real-Time PCR

The mRNA expression level of the Cullin7 molecule was analyzed by the Real-Time PCR (RT-PCR, real-time polymerase chain reaction) using qPCR SYBR Master Mix (Jena Bioscience Gmbh, Jena, Germany Cat no: PCR-372) commercial kit. The β-actin gene was used as the reference gene. The primers ACCTGAAGGCGGTCTCTGT and CCTTGCCATCTCGAATC were used as the forward and reverse primers, respectively. All procedures were performed in ice with a reaction volume of 20 µL. For the reaction mixture, 0.6 µL forward primer, 0.6 µL reverse primer, 6.8 µL PCR grade water, 10 µL qPCR SybrMasterMix, and 2 µL cDNA were prepared (Table 3). Afterward, the sample mixtures were placed into a 96-well plate and then placed in the Biorad CFX Connect device, and the reaction was carried out. After an initial denaturation at 95 °C for 2 min, the reaction consisted of 40 cycles of denaturation at 95 °C for 15 s, annealing at 52 °C for 1 min, and extension at 60 °C for 1 min.

### 2.3. Determination of Protein Levels

#### Enzyme-Linked Immunosorbent Assay (ELISA)

A commercial ELISA kit (BT LAB, Bioassay Technology Laboratory, Jiaxing Korain Biotech Co., Ltd., Jiaxing, China) was used to determine the Cullin7 protein levels in patient and control samples. Before this study, plasma and saliva samples were taken out of the −80 °C freezer and placed at room temperature. To determine Cullin7 levels, standards were prepared from 24 ng/mL stock standard at 12 ng/mL, 6 ng/mL, 3 ng/mL, 1.5 ng/mL, and 0.75 ng/mL using standard diluent solution. Plasma, saliva samples, and anti-Cul7 antibodies were added to the wells. The standard solution was added to the standard wells. Afterward, Streptavidin HRP was added to all wells and incubated at 37 °C for 60 min. Unbound antibodies were removed by washing. Substrate A and Substrate B solutions were added to all wells and incubated at 37 °C in the dark. Then, stop solution was added to all wells, and absorbance concentrations were measured using an ELISA reader read at 450 nm (Thermo ScientificTM MultiskanTM GO Microplate Spectrophotometer, Waltham, MA, USA).

### 2.4. Statistical Analysis

Statistical analyses were performed using SPSS Software (SPSS 22, Chicago, IL, USA). The variables were investigated using analytical methods (Kolmogorov–Smirnov/Shapiro–Wilk’s test) to determine whether or not they were normally distributed. As a result, the non-parametric Mann–Whitney U test was preferred to calculate statistical significance between parameters. The chi-square test was used to determine whether the difference between the observed frequencies and the expected frequencies was statistically significant. A *p*-value of less than 0.05 was considered to show a statistically significant result. The diagnostic values of the tests were analyzed using the ROC (Receiver Operating Characteristic) curve.

## 3. Results

### 3.1. Gene Expression Levels

Mean (x), standard deviation (sd), and median values (m) of plasma *Cul7* gene expression levels were calculated as 41.13 ± 80.41; 3.08 and 8.72 ± 14.85; 1.04 in breast cancer patients and healthy female controls, respectively. Although the plasma *Cul7* gene expression level was found to be higher in the patients compared to the healthy control group, it was statistically insignificant (*p* = 0.222).

The arithmetic mean, standard deviation, and median values of salivary *Cul7* gene expression levels were calculated as 10.1 ± 42.85; 0.66 in patients with breast cancer and 5.85 ± 17.7; 1.27 in controls. The salivary *Cul7* gene expression levels were found to be lower in patients compared to the control group and statistically insignificant (*p* = 0.876) (Table 4).

When the chi-square test was performed, the relationship between the patient’s age, ER, PR, HER2, histology, menopause status, plasma, and saliva gene expression levels of *Cul7* was statistically insignificant (*p* > 0.05).

**Figure 1:**
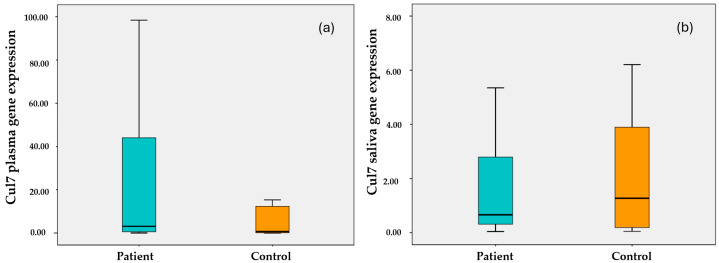
Gene expression of plasma (**a**) and saliva (**b**) *Cul7* median levels of breast cancer patients and healthy controls.

As seen in Figure 1a, plasma *Cul7* gene expression levels are above the median value (m = 3.08, m = 1.04, respectively) in breast cancer patients and healthy control groups. Also, salivary *Cul7* gene expression levels show a distribution above the median value (respectively; m = 0.66, m = 1.27) in breast cancer patients and healthy control groups (Figure 1b).

**Figure 2:**
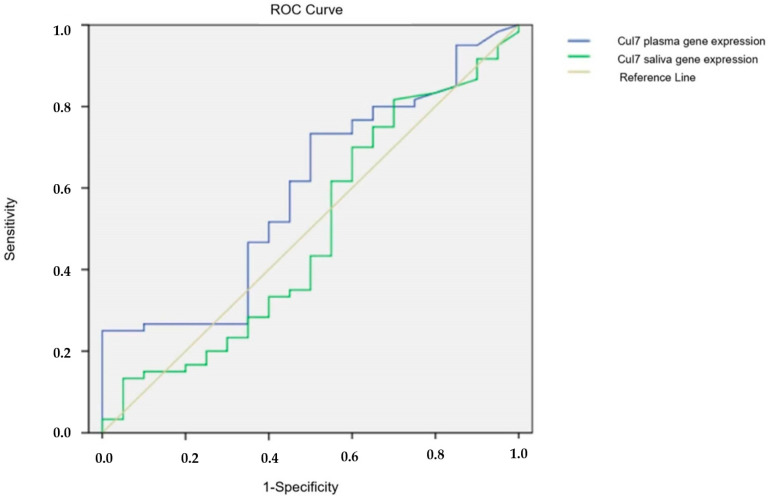
ROC analysis of gene expression tests.

For the *Cul7* gene expression test, the Area Under Curve (AUC) was determined by the ROC (Receiver Operating Characteristic) analysis. When the AUC values were calculated, it was determined that the diagnostic value of the tests was low in both plasma and saliva (AUC: 0.592, 95% confidence interval: 0.490–0.694; AUC: 0.488, 95% confidence interval: 0.386–0.589 respectively) (Figure 2).

### 3.2. Protein Levels

The mean (x), standard deviation (sd), and median (m) values of plasma protein Cul7 were found to be 4.93 ± 4.8; 3.12 ng/mL and 3.69 ± 2.48; 2.8 ng/mL in breast cancer patients and female healthy controls, respectively. The mean, standard deviation, and median values of salivary protein Cul7 were 2.72 ± 2.16 and 2.25 ng/mL in patients and 2.61 ± 1.36 and 1.9 ng/mL in controls. Although plasma and salivary protein Cul7 levels in breast cancer patients were higher than in the healthy control group, the difference was statistically insignificant (*p* = 0.463, *p* = 0.938) (Table 5).

Between the patients’ age, ER, PR, HER2, histology, menopause status, plasma, and saliva protein levels of Cul7 with the Chi-square test, a statistically insignificant difference was found.

**Figure 3:**
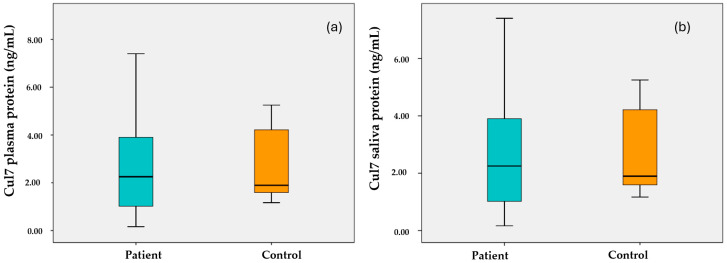
Box plots of Cul7 protein in plasma (**a**) and saliva (**b**) samples of breast cancer patients and healthy controls.

As seen in Figure 3a, plasma Cul7 protein levels are above the median value (m = 3.12, m = 2.8, respectively) in breast cancer patients and healthy control groups. As seen in Figure 3b, salivary Cul7 protein levels show a distribution close to the median value (m = 2.25) in patients with breast cancer, while it is above the median value (m = 1.9) in the healthy control group.

**Figure 4:**
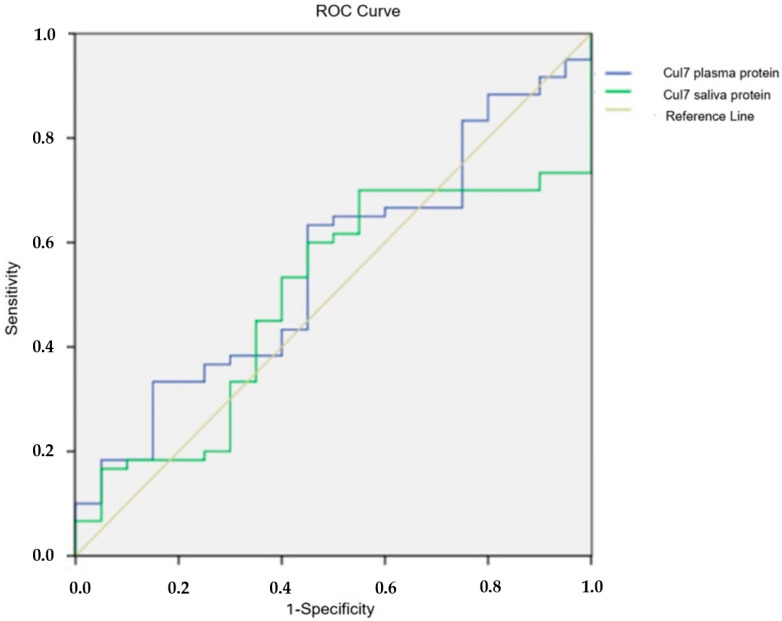
ROC analysis of plasma and saliva Cul7 protein tests.

For the Cul7 test, the Area Under Curve/AUC was determined by ROC (Receiver Operating Characteristic) analysis. When the AUC values were calculated, it was determined that the diagnostic value of the tests was low in both plasma and saliva (AUC: 0.555, 95% confidence interval: 0.415–0.695; AUC: 0.494, 95% confidence interval: 0.357–0.632 respectively) (Figure 4).

The expression levels of the *Cul7* molecule at both mRNA and protein levels in plasma and saliva samples of patients were analyzed by the Mann–Whitney U test. Based on this analysis, statistically significant relationships were identified among the following groups: plasma protein and saliva protein; plasma protein and saliva gene expression; saliva protein and saliva gene expression; plasma gene expression; and saliva gene expression (*p* = 0.000). Statistical significance values are given in Table 6.

## 4. Discussion

Breast cancer ranks first among cancer-related deaths in women. Due to genetic and epigenetic changes, breast cancer has various biological and histological features [1]. Because of its heterogeneous structure, different clinical findings are observed. Therefore, there are differences in the treatment to be applied and the response to the treatment, and, as a result, the prognosis uncertainty has caused the search for new biomarkers for breast cancer.

Numerous studies have shown that Cul family members were associated with the initiation and progression of tumors. Mutations or deregulation in several members of this gene family have been reported to be associated with the emergence of malignant phenotypes. These changes mainly affect substrate stability, which regulates complex signaling pathways in cells and controls cell proliferation and cell cycle progression, thereby promoting tumorigenesis or suppressing tumor growth/metastasis [16,17].

Studies published to date have not found any research investigating the effectiveness of Cul7 in saliva. Although our study is unique in this area, it is the first study in which the role of this molecule in breast cancer has been investigated together in both plasma and saliva.

In a 2013 study, the expression of Cul7 in hepatocellular carcinoma (HCC) cell lines and five different tissue samples was determined by quantitative reverse transcription PCR and Western blot method. In addition, protein expression of Cul7 was investigated in 162 HCC paraffin block tissue samples by immunohistochemistry, and it was reported that *Cul7* was a novel gene potentially associated with HCC pathogenesis and progression [18].

In 34 patients with HCC and seven different cell line samples analyzed by qRT-PCR and Western blot, *Cul7* showed high expression, especially in metastatic tumor tissues; there was a negative correlation between *Cul7* expression and long survival, and it was determined that migration, invasion, and metastatic properties were significantly reduced by silencing of *Cul7* in liver cancer cells. It has also been shown that Cul7 promoted epithelial–mesenchymal transformation of cancer cells. The results of this study helped to elucidate the oncogenic functions of Cul7 in liver cancer [19].

In the study by An J. et al. with 162 paraffin block tissue samples with HCC, *Cul7* was expressed in 69.1% of HCC tissues, while expression was observed in only 29% of adjacent normal hepatic tissues. The expression in HCC tissues was significantly higher than in adjacent normal hepatic tissues (*p* < 0.01), suggesting that *Cul7* might play a role in the pathogenesis of HCC [20].

Cul7, which acts as a scaffold protein in the E3 ligase complex, is thought to have a potential oncogenic role in pancreatic cancer by promoting the proliferation of pancreatic cancer cells [21].

In a study with choriocarcinoma tissue samples, it has been shown that the ZEB1 (Zinc Finger E-Box Binding Homeobox 1) and Slug (Snail Family Transcriptional Repressor 2) genes, the transcription repressor of E-cadherin, are significantly upregulated in JEG-3 cells overexpressing *Cul7*. How Cul7, an essential component of the RING E3 ligase, triggers the activation of these two transcriptional factors remains to be determined. Fu et al. suggested that Cul7 was a regulator of the endothelial–mesenchymal transition (EMT) in choriocarcinoma that had an important role in the invasion and metastasis of tumors [22].

In this study performed in breast cancer tissues, Cul7 protein levels were compared with normal breast tissues, and it was determined that Cul7 expression was significantly higher in breast cancer samples (*p* < 0.01 is based on Student’s *t*-test). Cul7 has been shown to promote breast cancer cell proliferation and invasion by downregulating p53 expression. Therefore, it is thought that Cul7 functions as a novel oncogene in breast cancer and provides evidence that it could be a potential therapeutic target [23].

In the immunohistochemical study conducted by Qiu et al. in 2018 with 13 normal, 20 benign, and 93 breast cancer tissues, it was shown that high *Cul7* expression was significantly associated with the pathological stage of breast cancer and lymph node metastasis. It has been noted that patients with high *Cul7* expressions had a shorter overall survival rate than those with low *Cul7* expressions [14].

Analysis of paraffin blocks of tissue samples from 120 epithelial ovarian cancer patients found that the expression level of Cul7 was significantly associated with tumor stage and lymph node metastasis in these patients and was a remarkable predictor of poor clinical prognosis [24].

In the immunohistochemistry study of 130 patients with esophageal cancer, the expression of *Cul7* in paraffin block tissue samples obtained was found to be significantly higher than in non-tumor tissues (*p* = 0.000). The χ^2^ analysis confirmed that *Cul7* expression was positively correlated with the depth of invasion (*p* = 0.000), lymph node involvement (*p* = 0.033), and advanced clinical stage (*p* = 0.000). Survival analysis showed that *Cul7* was positively associated with poor overall survival (*p* = 0.001) and disease-free survival (*p* = 0.0019) [25].

Men et al. found that increased Cul7 protein expression in lung cancer tissues and decreased expression of Cul7 expression in lung cancer cells inhibited cell proliferation and xenograft tumor growth [26].

High *Cul7* expression has been associated with high tumor grade, mesenchymal molecular glioma subtype, and poor prognosis in glioma patients. Silencing the *Cul7* gene in U87MG and U251 cells significantly inhibited tumor growth, invasion, and migration in vitro and in vivo. Western blot analysis performed under conditions where Cul7 was silenced revealed that cyclin-dependent kinase inhibitors and molecular markers of epithelial–mesenchymal transition (EMT) were altered. In contrast, Cul7 overexpression appeared to promote tumor growth, invasion, and migration. Gene set enrichment analysis (GSEA) and Western blot showed that Cul7 was positively associated with the NF-κB pathway [27].

As far as is known, Cul7 has been studied in various tumor types and has been found to play an important role in the initiation and development of various cancer types. It has revealed that Cul7 promoted cell growth, invasion, and metastasis in many of these tumors, might play an oncogenic role in cancer, and had the potential to be used as a biomarker for the prognosis of metastatic cancers.

In 2024, Zhang et al. investigated the role of Cullin family genes in colorectal cancer (CRC), and analysis in the UALCAN database showed that Cul7 was significantly upregulated in CRC patients compared to normal controls (*p*-value < 0.05). Similarly, analyses using the GEPIA2 database also yielded consistent results, and the upregulation of Cul7 in CRC tissues compared to normal tissues was confirmed. Then, the HPA database was used to examine the protein expression profiles of Cullin family genes in CRC, and immunohistochemistry-based results showed that Cul7 protein expression was higher in CRC tissue samples than in normal samples [28].

In addition to this research, there are studies showing that Cul7 may also have the opposite effect and suppress cell growth. DeCaprio et al. discovered a potential tumor suppressor role of Cul7 in viral transformation using an SV40 T antigen model [29]. It has also been reported that the mTOR/IRS-1 negative feedback loop was related to the inhibition of malignancy by limiting PI3K activity [30,31]. IRS-1, the critical molecule downstream of insulin and insulin-like growth factor 1 receptor, can be ubiquitinated by Cul7 E3 ligase, suggesting that this tumor-suppressing activity may be related to Cul7-mediated degradation of IRS-1. In 2018, Sun et al. revealed that CD36 could negatively regulate insulin activation; CD36 interacts with IRS-1 and, thus, abrogates the binding between IRS-1 and Cul7, which further increases IRS-1 stability, thereby affecting insulin signaling and ultimately resulting in tumor suppression [32].

In summary, CRLs (Cul Ring Ligases) are the largest family of E3 ligases, and their multiple substrates form Cul-based protein complexes for ubiquitination and degradation. Given the intricate role of cullins and CRLs in various biological processes, cullin dysfunction is anticipated to contribute to disease pathogenesis. Notably, mutations in Cul7 have already been linked to certain human disorders [33,34]. Cul7 is a member of the CRL that can have the dual effect of “degrading” or “not degrading” substrates. While *Cul7* exhibits increased expression in several types of cancer, it participates in tumor growth, invasion, and metastasis and is also associated with clinical stage and prognosis. Cul7 also exerts a tumor suppressor effect, as evidenced in part by the degradation of IRS-1 via the mTOR pathway. Additionally, while Cul7 performs non-proteolytic functions, it regulates its substrates without binding Fbxw8 or Rbx1. Therefore, future studies may focus on the function of Cul7 in the progression and metastasis of various types of cancer and the detailed mechanism that controls the interactions between Cul7 and its complexes. Thus, in light of the useful information obtained, it is thought that steps can be taken toward improving pharmaceutical drug development [35].

In our study, when Cul7 gene expression levels in plasma and saliva samples of breast cancer patients and healthy controls were compared, although the plasma samples were found to be higher in the patients than in the control group, statistical insignificance was detected between them (*p* > 0.05). While saliva gene expression levels were found to be higher than the control group patients, a statistically insignificant difference was detected (*p* > 0.05).

When the Cul7 protein levels in the plasma and saliva samples of the patient and control groups were compared, although the level of the patients was higher than the control group, no statistical significance was detected between them (*p* > 0.05).

No correlation was found between the patient’s age, ER, PR, HER2, histology, menopausal status characteristics, and plasma and saliva *Cul7* gene expression and protein levels (*p* > 0.05).

In this study, the potential of Cul7 to be used as a biomarker at the protein and mRNA levels was examined, and it was observed that the increase in expression in plasma was higher than in saliva. Therefore, plasma is thought to be a better study material for Cul7. Secondly, if saliva material is to be studied, the expression of *Cul7* at the mRNA level may be a better biomarker compared to the protein level. Approximately 99% of saliva consists of water, and the remaining 1% consists of serum components, inorganic ions, enzymes, and glycoproteins. Biomarkers can include proteins, carbohydrates, lipids, or microorganisms. Alterations in the composition of these biological molecules can indicate the progression of an underlying disease, aiding in its diagnosis, management, prognosis assessment, and outcome monitoring [36].

The serum elements it contains are obtained from the local vascular network of the carotid artery. Therefore, saliva is rich in many molecules in circulation and is an important body fluid for early diagnosis [37]. It is also known that saliva contains exosomes, which are small vesicles containing lipids, mRNA, micro-RNA, DNA, and proteins, and it is thought that exosomes carry these contents from distant places to the whole body [38,39,40,41,42]. Saliva is an easily accessible material that can be studied with noninvasive methods in the diagnosis and treatment evaluation of various diseases, as it can be easily obtained without the need for healthcare personnel and has a low cost.

The data obtained from our study are evaluated alongside information from the literature, showing that identifying Cul7 as a protein may be challenging due to potential posttranscriptional modifications occurring during its synthesis from mRNA to protein. Nevertheless, the findings of our study suggest that examining mRNA expression levels may offer valuable insights into the potential of Cul7 as a biomarker for early disease diagnosis. To our knowledge, this is the first study to investigate the Cul7 molecule in plasma and saliva at both the gene and protein levels. However, the small sample size of our study represents a limitation, and future studies with larger sample sizes are needed to validate and expand upon these findings.

## Figures and Tables

**Table 1:** Clinical characteristics of breast cancer patients.

Parameter	n	%
**Number of patients**	60	100
**Mean age (min–max)**	49.06 (27–77)	100
<49	32	53
≥49	28	47
**Histology**		
Invasive ductal carcinoma	47	79
Invasive lobular carcinoma	6	10
Lobular carcinoma in situ	2	3
Mucinous carcinoma	2	3
Infiltrative ductal carcinoma	1	2
Mixed types	2	3
**ER**		
+	55	92
−	5	8
**PR**		
+	50	83
−	10	17
**HER2**		
+	11	18
−	49	82
**Menopause**		
Pre	32	53
Post	28	47

**Table 2:** Reaction components for cDNA synthesis.

Reaction Components	1 Volume for 1 Sample (μL)
RNAse-free water	10.5 μL
Script RT Buffer	4 μL
dNTP mix	1 μL
Oligo dT Primer	0.5 μL
DTT	1 μL
RNAse Inhibitor	0.5 μL
RT Enzyme	0.5 μL
Total RNA	2 μL

**Table 3:** Real-Time PCR components.

RT PCR Reaction Components	Volume for 1 Sample
Primer F	0.6 µL
Primer R	0.6 µL
PCR grade water	6.8 µL
qPCR SybrMasterMix	10 µL
cDNA	2 µL
Total Volume	20 µL

**Table 4:** The statistical significance (*p*-values) of gene expressions with mean (x), standard deviation (sd), median (m), minimum (min), and maximum (max) values in breast cancer patients and female healthy control groups.

Gen Expression Levels	Breast Cancer (n = 60) x ± sd m (Min, Max)	Control (n = 20) x ± sd m (Min, Max)	*p*-Value
Plasma *Cul7*	41.13 ± 80.41 3.08 (0.01–384.8)	8.72 ± 14.85 1.04 (0.01–51.23)	*p* = 0.222
Saliva *Cul7*	10.1 ± 42.85 0.66 (0.04–320.28)	5.85 ± 17.7 1.27 (0.05–80.56)	*p* = 0.876

**Table 5:** Mean (x), standard deviation (sd), median (m), minimum (min), maximum (max) values, and statistical significance (*p*-values) of Cul7 protein levels in breast cancer patients and female healthy control groups.

Protein Levels (ng/mL)	Breast Cancer (n = 60) x ± sd m (Min, Max)	Control (n = 20) x ± sd m (Min, Max)	*p*-Value
Plasma Cul7	4.93 ± 4.8 3.12 (0.29–22.95)	3.69 ± 2.48 2.8 (1.98–13.35)	*p* = 0.463
Saliva Cul7	2.72 ± 2.16 2.25 (0.16–13.06)	2.61 ± 1.36 1.9 (1.17–5.25)	*p* = 0.938

**Table 6:** Statistical significance between patients’ tests.

Compared Tests	*p*-Value
Plasma Protein–Plasma Gene Expression	*p* = 0.332
Plasma Protein–Saliva Protein	*p* = 0.000
Plasma Protein–Saliva Gene Expression	*p* = 0.000
Saliva Protein–Saliva Gene Expression	*p* = 0.001
Saliva Protein–Plasma Gene Expression	*p* = 0.442
Plasma Gene Expression–Saliva Gene Expression	*p* = 0.011

## Data Availability

The original contributions presented in this study are included in this article. Further inquiries can be directed to the corresponding author.
